# Case-control study of metabolic syndrome and ovarian cancer in Chinese population

**DOI:** 10.1186/s12986-017-0176-4

**Published:** 2017-02-28

**Authors:** Ying Chen, Lei Zhang, Wenxin Liu, Ke Wang

**Affiliations:** 1 Department of Gynecologic Oncology, Tianjin Medical University Cancer Institute and Hospital, Huanhuxi Road, Hexi District, Tianjin, 300060 China; 2 Key Laboratory of Cancer Prevention and Therapy, Tianjin, 300060 China; 3 National Clinical Research Centre of Cancer, Tianjin, 300060 China

**Keywords:** Metabolic syndrome, Ovarian cancer, Diabetes, Hypertension

## Abstract

**Background:**

Recent studies have proved metabolic syndrome (MetS) was linked to cancer risks. However, few data has examined the relationship between MetS and epithelial ovarian cancer (EOC).

**Methods:**

We conducted a population-based case-control study in Tianjin Medical University Cancer Institute and Hospital, China (2010–2015) that enrolled 573 EOC patients and 1146 matched controls. Data were collected through in-person interviews, anthropometric measurement, and 8-h fasting bloods drawn. MetS was estimated by Chinese Diabetes Society (CDS) definition requiring presence of ≥3 of the following risk factors: 1) body mass index (BMI) ≥25.0 kg/m^2^,2) fasting plasma glucose ≥6.1 mmol/L or 2-h plasma glucose ≥ 7.8 mmol/L, 3) systolic blood pressure ≥140 mmHg or diastolic blood pressure ≥90 mmHg, 4) triglyceride (TG) ≥1.70 mmol/L or high-density lipoprotein cholesterol (HDL-C) < 1.0 mmol/L. Statistics were completed using chi-square tests and logistic regression analysis. The survival analysis was conducted by the Kaplan-Meier method and Cox proportional hazard regression models.

**Results:**

MetS was significantly more prevalent among EOC (25.13%) than controls (6.89%). A statistically significant increase risk for EOC was observed for MetS (multivariable-adjusted OR = 3.187; 95% CI: 2.135–4.756). MetS was significantly associated with histological grade (*P < 0.001)*, FIGO stage (*P = 0.003*), and lymph node (LN) status (*P = 0.002*) of EOC. In binary logistic regression analysis, the presence of MetS predicts the risk of advanced FIGO stage (OR = 2.155, 95% CI: 1.327–3.498, *P* = 0.002), lower differentiation (OR = 2.472, 95% CI: 1.164–5.250, *P* = 0.019), and LN metastasis (OR = 2.590, 95% CI: 1.089–6.160, *P* = 0.031) of EOC. Moreover, MetS is the independent factor for the evaluation of PFS and OS of EOC patients (both of them *P < 0.001*) in Cox proportional hazard model.

**Conclusion:**

MetS is obviously related to increased EOC risk. EOC patients with MetS in Chinese population were found to have statistically significant tumor advanced stage, low differentiation, LN metastasis and poor prognosis.

## Background

Approximately 95% of ovarian cancers are of epithelial origin. Epithelial ovarian cancer (EOC) was the leading killer among women with gynecologic cancers. In 2015, there were 22,280 estimated new diagnoses of ovarian cancer and 14,240 deaths from the disease [1]. Statistic revealed the morbidity and mortality of ovarian cancer were rising obviously [2]. However, scientists do not reach a consensus about the prevalence of ovarian cancer because of oncologic diseases have multiple causes. Recently, many researchers considered tumorigenesis process in the body as a systemic disease [3]. So, research attentions focused on the etiology and cause of cancer that lead to dysfunction and abnormality of metabolism increasingly [4, 5].

The metabolic syndrome (MetS) is a cluster of risk factors that includes central adiposity, high blood pressure, elevated blood glucose levels, elevated triglycerides (TG), and low high-density lipoprotein cholesterol (HDL-C) [6, 7]. In the last several years, several interesting studies have been published showing an association between cancer risk and the different components of MetS [8]. A noted large population-based enrolled 16,677 participants who were on medications for hyperlipidemia, diabetes and hypertension and were followed them for up to 8 years. A total of 823 incidents of cancer occurred during the study period, including a significantly increased risk of pancreatic cancer in males and colorectal cancer in females. Additionally, risks of women with liver, gallbladder and billiard tract, breast, and endometrial cancers were also increased [9]. MetS has emerged as a possible clinical condition that predisposes women to suffer breast and endometrial cancers, which associated with hormone disorder [10, 11].

However, epidemiologic studies linking MetS to ovarian cancer are scarce in spite that ovarian cancer is hormone related. Therefore, this present study aimed to collect the information on different components of MetS in a population-based control study of ovarian cancer and examined the role of metabolic dysfunction in EOC, in addition to examining risk with individual components of the MetS.

## Methods

### Study population

Our population-based case-control study of physical activity and ovarian cancer risk was approved by institutional review board of Tianjin medical university cancer institute and hospital. The clinicopathologic information of ovarian cancer group was collected from consenting patients diagnosed and treated for EOC between January 2010 and December 2015 at Department of Gynecologic Oncology, Tianjin Medical University Cancer Institute and Hospital. Clinical data from 630 consecutive EOC patients were extracted with routine preoperative serum detection. Twenty-five patients with concomitant endometrial cancer were excluded due to possible confounding neoplastic effect on serum lipid, while 32 patients were excluded with a previous history of cancer (five patients with breast cancer, seven with colon cancer, six with rectum cancer and fourteen with other cancers), leaving 573 patients for further analysis. The population-based controls were collected from Physical Examination Center, Tianjin medical university cancer institute and hospital, with all of the participants agreeing and signing consent forms. The controls had no history of hysterectomy, ovarian diseases, or previous cancer and were frequency matched to cases (2:1 ratio). Remarkably, there were not statistically different significances between the EOC group and the control group on age, pregnant times, menopause age, ever hormone use, and age of first pregnancy when choosing matched control cases.

### Data collection

Data were collected through in-person interviews using a structured questionnaire and cognitive interviewing methods, in which information on demographic variables and ovarian cancer risk factors including medical history and exogenous hormone use. Three measurements of height, weight and waist circumference were taken using standardized methods for anthropometric measurements at the time of interview, with the mean used as the final measurement. Blood was collected after a minimum 8-h fast, either prior to surgical treatment by hysterectomy or post surgery and subsequent to interviews for cases whose blood could not drawn pre-surgery. Blood was drawn post-interview among controls. A 10-mL blood sample was collected according to a standardized protocol, and samples were processed into blood fractions (serum, plasma, red blood cells, and buff coat), frozen at −80 °C within 24 h of collection, and transported for storage to a specimen bio-repository at the Department of Gynecological Oncology, Tianjin medical university cancer institute and hospital, Tianjin, China.

At present, there are two kinds of international definitions to diagnose MetS that are currently available for clinical use: (1) the National Cholesterol Education Program (NCEP)-Adult Treatment Panel (ATP) III [12]; (2) the International Diabetes Federation (IDF) [13]. Considering Chinese population was enrolled in this study, MetS was defined according to the Chinese Diabetes Society (CDS) definition [14]. Patients were diagnosed with MetS when they had three or more of the following indications: 1) body mass index (BMI) ≥25.0 kg/m^2^,2) fasting plasma glucose ≥6.1 mmol/L or 2-h plasma glucose ≥ 7.8 mmol/L, 3) systolic blood pressure ≥140 mmHg or diastolic blood pressure ≥90 mmHg, 4) triglyceride (TG) ≥1.70 mmol/L or high-density lipoprotein cholesterol (HDL-C) < 1.0 mmol/L. Participants met the criteria for high blood pressure or high fasting glucose concentration if they underwent hypertension or hyperglycemia treatment. BMI was calculated as weight in kilograms divided by the square of height in meters.

### Follow up

Data were collected until death or December 2016. Overall survival (OS) was defined as the time interval from the date of primary surgery to the date of death (failure) or to the end of follow-up for women who were alive (censored). Progression-free survival (PFS) was defined as the time elapsed from the date of primary surgery to the appearance of disease recurrence or progression (failure) or the last follow-up for women who were alive with no evidence of disease recurrence or progression (censored).

### Statistical analysis

Continuous data and frequency data were analyzed by Fisher’s exact test and the chi-square test. Results of continuous variables were expressed as mean ± standard deviation (SD). Logistic regression analysis was used to estimated ORs and 95% CIs for developing ovarian cancer in association with presence of MetS and individual biological MetS components. The individual biological MetS components were modeled as meeting the respective cut-point according to CDS definition. Two-sided *P*-values were considered statistically significant at *P* ≤ 0.05. The survival was determined by the Kaplan-Meier method, and the log rank test was used to determine significance. MetS and its components were included in the multivariate analysis by using of Cox proportional hazard regression models. Statistical analysis was performed using SPSS software passage for Windows (version 20.0; SPSS Inc., Chicago, IL, USA).

## Results

The participant characteristics in this study were presented in the Table 1. Among this population, the average ages in 573 EOC and 1146 control cases were 52.59 ± 9.20 and 52.97 ± 9.73 years, respectively. In Table 1, the proportion of cases with levels of TG, HDL-C, BMI were demonstrated in EOC and control groups according to the cut-offs for MetS criterion in China. The proportion of cases with a history of hypertension or diabetes was also collected in Table 1.

**Table 1:** characteristics of epithelial ovarian cancer cases and population-based controls

Characteristic	Epithelia ovarian cancer (*n* = 573)	Control (*n* = 1146)
	Case (n, %)	Case (n, %)
Age(mean ± SD), y	52.59 ± 9.20	52.97 ± 9.73
Pregnant times		
0	131 (22.86)	128 (11.17)
1-2	260 (45.38)	604 (52.71)
> 2	183 (31.94)	414 (36.13)
Menopause age(mean ± SD), y	50.9 ± 7.13	50.1 ± 7.82
Menopause		
yes	389 (67.89)	534 (46.60)
No	184 (32.11)	612 (53.40)
Ever hormone use	145 (25.30)	328 (28.62)
Estrogen only	14 (2.44)	23 (2.01)
Estrogen + progestin	103 (17.98)	273 (23.82)
Other hormone therapy	28 (4.89)	32 (2.79)
Age of first pregnancy	23.59 ± 4.20	23.87 ± 4.65
Fasting plasma glucose (mmol/L)	5.74 ± 1.75	5.52 ± 1.07
Diabetes history (cases)		
Yes	161 (28.10)	148 (12.91)
No	412 (71.90)	998 (87.09)
Body mass index, kg/m^2^	25.29 ± 3.52	24.18 ± 3.78
BMI ≥25.0 kg/m2	219 (38.22)	463 (40.40)
BMI*<*25.0 kg/m2	354 (61.78)	683 (59.60)
Weight(mean ± SD), kg	62.95 ± 9.3	61.70 ± 8.90
Waist circumference(mean ± SD), cm	81.02 ± 9.97	80.09 ± 9.36
Triglyceride (TG, mmol/L)	2.34 ± 1.42	1.92 ± 0.94
TG*<*1.70	170 (29.67)	544 (47.47)
TG ≥ 1.70	403 (70.33)	602 (52.53)
HDL-C (mmol/L)	1.57 ± 0.31	1.87 ± 0.39
HDL-C*<*1.0	92 (16.06)	54 (4.71)
HDL-C ≥ 1.0	481 (83.94)	1092 (95.29)
Hypertension history		
Yes	156 (27.23)	164 (14.31)
No	417 (72.77)	982 (85.69)

As given in Table 2, we compared the proportion of participants having MetS according to three different definitions and the results did not differ significantly. The kappa value of interrater agreement was 92.5% between CDS and ATP III, 93.2% between CDS and IDF, and 90.0% between ATP III and IDF. The prevalence of MetS in our whole population ranged from 12.62% to 13.90% overall, assessing by three MetS criterions respectively. A higher range in proportion of 24.96% to 27.75% among EOC patients was found according to MetS diagnosis compared to control population ranging from 6.46% to 6.98% (Table 2).

**Table 2:** proportion of metabolic syndrome by three different criteria in our study

Definition risk factors	Metabolic syndrome definition		
	NCEP-ATPIII	IDF	CDS
Waist circumference(WC)	>88 cm	>80 cm	NA
BMI	NA	NA	≥25.0 kg/m^2^
TG	≥1.69 mmol/L	≥1.70 mmol/L	≥1.70 mmol/L
HDL-C	<1.3 mmol/L	<1.3 mmol/L	<1.0 mmol/L
hypertension	Systolic BP ≥ 130 or diastolic BP ≥ 85 mmHg	Systolic BP ≥ 130 or diastolic BP ≥ 85 mmHg	Systolic BP ≥ 140 or diastolic BP ≥ 90 mmHg
Fasting blood glucose	≥6.1 mmol/L	≥5.6 mmol/L	≥6.1 mmol/L or 2-h plasma glucose ≥7.8 mmol/L
Criteria	3 or more of the above	WC necessary and any 2 or the above	3 or more of the above
Cases (%)			
Ovarian cancer	159 (27.75)	143 (24.96)	144 (25.13)
Control	80 (6.98)	74 (6.46)	79 (6.89)
Total	239 (13.90)	217 (12.62)	223 (12.97)
Kappa value			
CDS	92.5%	93.2%	
IDF	90.0%		

*NECP* national cholesterol education program, *ATPIII* adult treatment panelIII, *IDF* international diabetes federation, *CDS* Chinese diabetes society, *NA* not available

As shown in Table 3, the proportion of patients with MetS as identified by CDS guidelines was significantly greater among 144 cases (25.13%) than 79 control cases (6.89%) and was associated with a 3.187-fold increase in EOC risk (multivariable-adjusted OR = 3.187; 95% CI: 2.135–4.756). Similarly, the magnitude of the risk increase was also observed with the other 2 versions of MetS (ATPIIIand IDF), with statistically significant ORs ranging from 3.277 (95% CI: 2.150–4.993) to 3.376 (95% CI: 2.271–5.018) for the multivariable model (Table 3). EOC risk also was enhanced by most of the individual components of the MetS, including BMI ≥ 25.0 kg/m^2^ (multivariable-adjusted OR = 1.385; 95% CI: 1.129–1.699), TG ≥1.70 mmol/L (multivariable-adjusted OR = 2.861; 95% CI: 1.040–7.873), HDL-C < 1.0 mmol/L (multivariable-adjusted OR = 2.142; 95% CI: 1.730–2.652), ever being diagnosed and treated for hypertension (multivariable-adjusted OR = 2.423; 95% CI: 1.963–1.2.990), and diabetes (multivariable-adjusted OR = 2.240; 95% CI: 1.749–2.869). All of the above were *P* < 0.01*.*

**Table 3:** age-adjusted and multivariable ORs and 95% CIs for risk of ovarian cancer

Component	Ovarian cancer Case (%)	Controls Case (%)	Age-adjusted OR (95% CI)	Multivariable-adjusted^a^OR (95% CI)
BMI ≥25.0 kg/m^2^	219 (38.22)	463 (40.40)	1.609 (1.298–1.994)	1.385 (1.129–1.699)
TG ≥ 1.70 mmol/L	403 (70.33)	602 (52.53)	2.130 (1.720–2.639)	2.861 (1.040–7.873)
HDL-C < 1.0 mmol/L	92 (16.06)	54 (4.71)	3.807 (2.673–5.423)	2.142 (1.730–2.652)
Hypertension	156 (27.23)	164 (14.31)	2.396 (1.86403.081)	2.423 (1.963–2.990)
Diabetes	161 (28.10)	148 (12.91)	2.673 (2.007–3.439)	2.240 (1.749–2.869)
ATPIII	159 (27.75)	80 (6.98)	5.503 (4.092–7.399)	3.277 (2.150–4.993)
IDF	143 (24.96)	74 (6.45)	5.254 (3.864–7.144)	3.376 (2.271–5.018)
CDS	144 (25.13)	79 (6.89)	4.884 (3.615–6.598)	3.187 (2.135–4.756)

^a^Multivariable-adjusted model: The individual components of the metabolic syndrome have been mutually adjusted, menopause, pregnant times, and ever hormone use

Consequently, we compared the pathological characteristics between EOC patients with or without MetS as defined by definition of CDS in Table 4. One hundred and forty-four cases (25.13%) EOC patients were diagnosed with MetS using by definition of CDS. The mean age with MetS group was 56.02 ± 8.00 years, which was higher than the non-MetS group (51.44 ± 9.29 years). Among 144 patients with MetS, we found 50 cases (34.72%) with lower differentiation, 119 cases (82.64%) with advanced FIGO stage, and 33 cases (22.92%) with lymph nodes (LN) metastasis, respectively, which were obviously higher than non-MetS patients with lower differentiation (18.82%), advanced stage (69.93%), and LN metastasis (12.35%). According to our results, statistically significant differences were observed in tumor differentiation grade, FIGO stage, and LN status between patients with or without MetS (*P<0.05*). In other words, tumor combining with MetS was more malignant clinical pathological behaviors in EOC patients.

**Table 4:** comparison of pathological characteristics between ovarian cancer patients with or without metabolic syndrome using Chinese Diabetes Society definition

Variable	MetS (n, %)	Non-MetS (n, %)	*P*-value
Cases	144 (25.13)	429(74.87)	
Age(mean ± SD) (years)	56.02 ± 8.00	51.44 ± 9.29	*<*0.001
Histology			0.411
serous	100(69.44)	304(70.86)	
mucous and others	44(30.56)	125(29.14)	
Differentiation			*<*0.001
G1-2	94(65.28)	348(81.12)	
G3	50(34.72)	81(18.82)	
FIGO Stage			0.003
I-II	25 (17.36)	129(30.07)	
III-IV	119(82.64)	300(69.93)	
Lymph nodes metastasis			0.002
No	111(77.08)	376(87.65)	
Yes	33(22.92)	53(12.35)	

*MetS* metabolic syndrome, *SD* standard deviation, *FIGO* international federation of gynecology and obstetrics

Consequently, in age-adjusted binary logistic regression analysis, the presence of MetS predicts the risk of advanced FIGO stage (OR = 2.155, 95% CI: 1.327–3.498, *P* = 0.002), lower differentiation (OR = 2.472, 95% CI: 1.164–5.250, *P* = 0.019), and LN metastasis (OR = 2.590, 95% CI: 1.089–6.160, *P* = 0.031) of EOC patients (Table 5). Additionally, other parameters relating to MetS were listed in Table 5.

**Table 5:** Binary logistic regression analysis examining patients with MetS for characteristics of epithelial ovarian cancer

Variable	FIGO stage		OR (95% CI)^a^	*P*-value	Grade		OR (95% CI)^a^	*P*-value	LN metastasis		OR (95% CI)^a^	*P*-value
	I-II	III-IV			G_1–2_	G_3_			Yes	No		
MetS			2.155(1.327–3.498)	0.002			2.472(1.164–5.250)	0.019	33	111	2.590(1.089–6.160)	0.031
Yes	25	119			94	50			53	376		
No	129	300			348	81						
BMI(kg/m2)			2.089(1.241–3.516)	0.006			0.853(0.516–1.409)	0.534	34	175	0.777(0.435–1.388)	0.394
≥25	39	170			156	53			52	312		
<25	115	249			286	78						
DM			0.726(0.341–1.545)	0.406			0.819(0.485–1.383)	0.456	31	130	1.091(0.611–1.948)	0.769
Yes	29	132			121	40			55	357		
No	125	287			321	91						
HBP			1.576(0.969–2.564)	0.067			0.957(0.583–1.571)	0.862	32	144	0.971(0.553–1.705)	0.918
Yes	37	139			129	47			54	343		
No	117	280			313	84						
TG (mmol/L)			1.285(0.827–1.997)	0.266			1.386(0.829–2.316)	0.213	26	144	0.697(0.394–1.233)	0.215
TG*<*1.70	50	120			143	27			60	343		
TG ≥ 1.70	104	299			299	104						
HDL-C(mmol/L)			1.357(0.741–2.488)	0.323			1.014(0.566–1.816)	0.963	68	413	1.061(0.539–2.089)	0.864
HDL-C ≥ 1.0	133	348			378	103			18	74		
HDL-C*<*1.0	21	71			64	28						

*MetS* metabolic syndrome, *OR* odds ratio, *CI* confidence interval, *BMI* body mass index, *DM* diabetes mellitus, *HBP* high blood pressure, *TG* Triglyceride, *HDL-C* high-density lipoprotein cholesterol

^a^Adjusted for age in logistic regression model

**Fig. 1:**
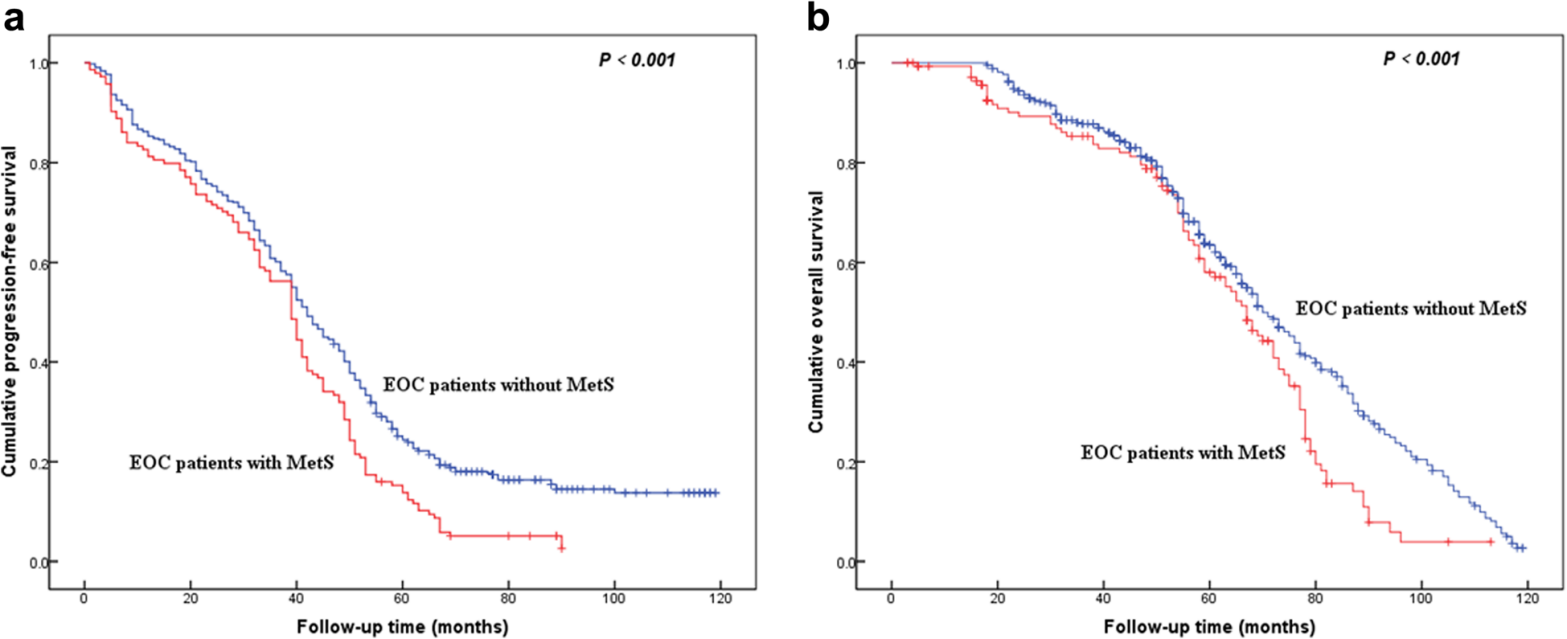
Kaplan–Meier curves for survival of 573 patients with epithelial ovarian cancer. Cumulative progression-free survival (**a**) and overall survival (**b**)

The survival analysis was showed in Table 6. By the Kaplan-Meier method of univariate analysis, the shorter median of PFS and OS were related to EOC patients with MetS (39 *vs* 42 months and 67 *vs* 71 months, respectively, both of them *P < 0.01*, Fig. 1) and BMI ≥25 kg/m^2^ (40 *vs* 44 months and 67 *vs* 70 months, respectively, both of them *P < 0.01*). Furthermore, in Cox proportional hazard model, MetS was the independent factor for the evaluation of PFS and OS of EOC patients (both of them *P < 0.001*).

**Table 6:** Univariate and multivariate survival analysis of MetS for progression-free and overall survival in 573 EOC patients

Variable	Cases (N)	Progression-free survival (PFS)					Overall survival (OS)				
		Univariate analysis		Multivariate analysis			Univariate analysis		Multivariate analysis		
		Media of PFS	*P* ^*a*^	OR	95% CI For HR	*P* ^*b*^	Media of OS	*P* ^*a*^	OR	95% CI For HR	*P* ^*b*^
MetS			*<*0.001	1.482	1.087–2.022	0.013		*<*0.001	1.702	1.195–2.424	0.003
Yes	144	39					67				
No	429	42					71				
BMI(kg/m2)			0.006	0.926	0.746–1.150	0.486		0.002	0.873	0.678–1.123	0.290
≥25	209	40					67				
<25	364	44					70				
DM			0.071	1.087	0.883–1.338	0.430		0.077	1.096	0.850–1.413	0.480
Yes	161	40					69				
No	412	41					70				
HBP			0.074	0.992	0.789–1.247	0.942		0.753	0.778	0.594–1.019	0.068
Yes	176	39					69				
No	397	42					72				
TG (mmol/L)			0.556	0.954	0.773–1.178	0.661		0.451	0.949	0.737–1.221	0.682
TG*<*1.70	170	41					70				
TG ≥ 1.70	403	41					69				
HDL-C(mmol/L)			0.286	1.024	0.796–1.319	0.852		0.097	1.115	0.839–1.481	0.454
HDL-C ≥ 1.0	481	41					70				
HDL-C*<*1.0	92	40					67				

*MetS* metabolic syndrome, *P* ^*a*^ *P* value, log rank test, *OR* odds ratio, *CI* confidence interval, *P* ^*b*^ *P* value, Cox regression

## Discussion

MetS was originally recognized as a cluster of risk factors that better predicted cardiovascular disease and diabetes incidence, than simple BMI or obesity measures [15] since it was firstly proposed by Reavan in 1988 [16] and the accepted criteria for clinical identification of the components of MetS has been promulgated by NCP-ATPIII [17] and WHO as well as IDF [13], and the American Association of Clinical Endocrinologists (AACE) [18]. At present, accumulating epidemiological literature appeared and had manifested that MetS was closely related to the occurrence and development of malignant diseases in different territorial populaiton [8]. Chiu HM et al.[19] and Morita T et al. [20] had reported people with MetS are at increased risk of colon cancer and adenoma in Asian populations. Sha N et al.[21] also observed that MetS was significantly associated with histological grade and stage of bladder cancer in 323 patients of Chinese population. Especially for endometrial cancer, collective data supported MetS could be a means for identifying a risk of endometrial cancer that might otherwise be missed or before any one component of MetS becomes more advanced [7]. Ni et al.[22] also clarified that MetS is associated with FIGO stage, grade, vascular invasion, tumor size, and lymphatic metastasis in endometrial cancer and confirmed MetS lead to a poor outcome in Chinese patients with endometrial cancer. A case–control study from Italian population revealed that MetS definition most strongly associated with endometrial cancer included BMI >30 kg/m2 and at least 2 of hypertension, diabetes, and hyperlipidemia [23]. Furthermore, a study in Norway suggested that inactivity and high energy intake are major risk factors for endometrial cancer [24].

However, limited study was available on the relationship between MetS, as well as the components of MetS, and characteristics of EOC. Thus, we designed this population based case-control study of EOC to explore the association between MetS, as well as components of MetS, and several important clinical characteristics and prognosis of EOC patients.

To begin with, 573 EOC and 1146 control cases were included in this study according to the case-control matching standard. The judgment of MetS and further analysis with EOC were reference to CDS definition. So, we firstly evaluated the consistency incidence of MetS estimation among CDS, ATP III and IDF definitions. Statistics demonstrated the kappa value of interrater agreement was 92.5% between CDS and ATP III, 93.2% between CDS and IDF, and 90.0% between ATP III and IDF, which indicated that CDS definition was available and a little superior to the international admissive criterions in our Chinese population. Additionally, the case proportion of MetS in EOC patients was found to be higher than the control population whichever assessing by three MetS criterions respectively.

Consequently, in logistic regression model, we found 3 different definitions of MetS, as well as the components of MetS, were all associated with an elevated EOC risk. the proportion of patients with MetS as identified by CDS guidelines was significantly greater among 144 cases (25.13%) than 79 control cases (6.89%) and was associated with a 3.187-fold increase in EOC risk.

Previously, epidemic literature about MetS as a cluster of risk assessed with regard to ovarian cancer in large scale project was very scary only in one study. The research conducted by Bjorge and colleagues [25] included 287,320 women from Austria, Norway and Sweden. Relative risks of ovarian cancer were estimated using Cox regression from MetS. Their results suggested 644 EOC and 388 deaths from ovarian cancer were identified during follow-up. In the end, they concluded there was no overall association between MetS and ovarian cancer risk. However, increasing levels of cholesterol and blood pressure increased the risks of mucinous and endometrioid tumors, respectively. Increasing levels of BMI conferred an increased risk of ovarian cancer mortality in women above the age of 50 years. There are a few unconformities between Bjorge’s and our conclusions, which may be contributed to the different race population and study design. In order to avoid bias and deviations, more and further studies of multi-centers deserved to perform to explore the relationship between MetS and ovarian cancer.

Furthermore, we compared the pathological characteristics between EOC patients with or without MetS as defined by definition of CDS. Statistics significantly proved the differences were observed in tumor differentiation grade, FIGO stage, and LN status between patients with or without MetS, which implied that tumor combining with MetS was more malignant clinical pathological behaviors in EOC patients. What’s more, in binary logistic regression analysis, the presence of MetS predicts the risk of advanced FIGO stage, lower differentiation, and LN metastasis of EOC patients.

As we know, based on symptoms before the development of ovarian cancer, such as irregular menstruation and then amenorrhea, and overweight, there is an assumption that polycystic ovaries syndrome (PCOS) can precede ovarian cancer [26]. Importantly, one of the criteria for PCOS is overweight or obesity. Obesity and MetS are constant companions of PCOS [27].

Obesity is one of the characteristics of MetS, according to the population-based longitudinal study in the People’s Republic of China. Standardized prevalence has reached up to 9.1% for obese population [28]. Notably, it has already accounted for a significant proportion. A number of studies verified obesity increased risk of several cancers, including breast, endometrium, kidney, esophageal, bladder, and colon carcinomas [29]. Especially, Calle et al. had already proved that significant trends of increasing risk with higher BMI values were observed for death of many cancers, including ovarian cancer. Importantly, they concluded increased body weight was associated with increased death rates for all cancers combined and for cancers at multiple specific sites [30]. Similarly, studies showed the relationship between the development of neoplastic diseases of female genitals (ovary and uterus) and presence of obesity [31, 32]. Approximately 60% to 90% of patients with ovarian cancer and endometrial cancer have overweight [33, 34]. Some studies have indicated obesity is a negative prognostic indicator for survival [35]. Large cohorts of ovarian cancer patients have demonstrated that the risk of ovarian cancer mortality is increased among those with higher BMI [36, 37]. A BMI ≥ 25 kg/m^2^ was used as measurement for obesity in our study. Our results showed BMI is associated with FIGO stage of ovarian cancer in binary logistic regression analysis. Some reasons may be used to explain the result. The cancer cells use the glucose, fatty acids, ketones, lactate, cholesterol, and other metabolites of fats and carbohydrates metabolism [38]. Biochemically, excess energy in hosts can contribute to risks of carcinogenesis [39, 40]. Excessive fat is also associated with systemic inflammatory response, which may play an important role in cancer. Interestingly, Oshakbayev et al.[41] ever reported a case that they treated a 41-year-old woman with end-stage ovarian carcinoma by using of weight loss therapy. While the patient was losing the gained body mass, tumors surprisingly shrank or disappeared (ultrasound data) during the observation period after start of the treatment.

Additionally, studies had already illustrated that diabetes was an independent risk factor for mortality in patients with EOC [42]. Ovarian cancer patients with diabetes were found to have a two and a half year lifespan reduction compared to non-diabetes [43]. Possible reason maybe reduced insulin sensitivity and elevated levels of IGF-1 [21]. IGF-1 is a growth factor that is secreted by the liver and is commonly associated with obesity and hyperinsulinism [44]. Hyperinsulinism decreases hepatic secretion of IGF binding protein (IGFBP), further increasing evels of free IGF-1 [42]. Conversely, starvation and calorie restriction are associated with lower levels of IGF-1 and downstream signaling [42]. IGF-1 has been confirmed to enhance growth of ovarian cancer cell [45]. Furthermore, previous research had proved that the high expression of IGF-1 in obesity and DM indicated the increased risk of EOC and poor prognosis [42].

According to our data, the components of MetS (diabetes, hypertension, TG, and HDL-C), when assessed individually, it was no statistically significant associated with advanced stage, low differentiation, and LN metastasis of EOC. However, when they considered with MetS, patients with MetS were found to have statistically significant advanced stage, low grade and LN metastasis of EOC.

Finally, we also proved that MetS was the independent factor for the evaluation of PFS and OS of EOC patients in Cox proportional hazard model, which were consistent with previous results in other cancers. Ni et al. showed that MetS was an independent prognostic factor for endometrial adenocarcinoma [22]. Voutsadakis reviewed published literature and indicated that obesity and diabetes were the prognostic factors in colorectal cancer [46].

## Conclusion

Conclusively, MetS criteria of CDS are applicable and appropriate in Chinese population. Our study provides strong evidence for a role of MetS in EOC risk. EOC risk increases with presence of MetS compared to the control Chinese population. EOC patients with MetS were found to have statistically significant advanced FIGO stage, low tumor grade, and LN metastasis. Furthermore, the presence of MetS predicts the risk of advanced FIGO stage, lower differentiation, and lymph node metastasis of EOC patients. Moreover, MetS is the independent indicator for the PFS and OS evaluations of EOC patients. Thus, possible recommendations to reduce ovarian cancer should continue to encourage women to maintain a healthy weight and targeting MetS maybe reduce the EOC risk. Of course, further study certainly should be taken to confirm our results in the future.

## Abbreviations

BMI: Body mass index
CDS: Chinese Diabetes Society
EOC: Epithelial ovarian cancer
FIGO: International federation of gynecology and obstetrics
HDL-C: High-density lipoprotein cholesterol
LN: Lymph node
MetS: Metabolic syndrome
TG: Triglyceride

## References

[1] Siegel RL, Miller KD, Jemal A (2016). Cancer statistics, 2016. CA Cancer J Clin.

[2] Chen W (2016). Cancer statistics in China, 2015. CA Cancer J Clin.

[3] Aituov B (2012). Pathogen-driven gastrointestinal cancers: Time for a change in treatment paradigm?. Infect Agent Cancer.

[4] Balogun N (2012). Noninvasive nutritional management of ovarian cancer patients: beyond intestinal obstruction. Int J Gynecol Cancer.

[5] Extermann M (2013). Metabolic syndrome and cancer: from bedside to bench and back. Interdiscip Top Gerontol.

[6] Micucci C (2016). Current perspectives between metabolic syndrome and cancer. Oncotarget.

[7] Friedenreich CM (2011). Case**-**control study of the metabolic syndrome and metabolic risk factors for endometrial cancer. Cancer Epidemiol Biomarkers Prev.

[8] Uzunlulu M, Telci Caklili O, Oguz A (2016). Association between metabolic syndrome and cancer. Ann Nutr Metab.

[9] Russo A, Autelitano M, Bisanti L (2008). Metabolic syndrome and cancer risk. Eur J Cancer.

[10] Trabert B (2015). Metabolic syndrome and risk of endometrial cancer in the united states: a study in the SEER-medicare linked database. Cancer Epidemiol Biomarkers Prev.

[11] Bhandari R (2014). Metabolic syndrome is associated with increased breast cancer risk: a systematic review with meta-analysis. Int J Breast Cancer.

[12] National Cholesterol Education Program Expert Panel on Detection, E. and A. Treatment of High Blood Cholesterol (2002). Third Report of the National Cholesterol Education Program (NCEP) Expert Panel on Detection, Evaluation, and Treatment of High Blood Cholesterol in Adults (Adult Treatment Panel III) final report. Circulation.

[13] Alberti KG, Zimmet P, Shaw J (2006). Metabolic syndrome--a new world-wide definition. A Consensus Statement from the International Diabetes Federation. Diabet Med.

[14] Zhou H (2010). Evidence on the applicability of the ATPIII, IDF and CDS metabolic syndrome diagnostic criteria to identify CVD and T2DM in the Chinese population from a 6.3-year cohort study in mid-eastern China. Diabetes Res Clin Pract.

[15] Expert Panel on Detection, E. and A. Treatment of High Blood Cholesterol (2001). Executive Summary of The Third Report of The National Cholesterol Education Program (NCEP) Expert Panel on Detection, Evaluation, And Treatment of High Blood Cholesterol In Adults (Adult Treatment Panel III). JAMA.

[16] Reaven GM (1988). Banting lecture 1988. Role of insulin resistance in human disease. Diabetes.

[17] Lakka HM (2002). The metabolic syndrome and total and cardiovascular disease mortality in middle-aged men. JAMA.

[18] Ford ES (2003). Insulin resistance syndrome: the public health challenge. Endocr Pract.

[19] Chiu HM (2015). Effects of metabolic syndrome and findings from baseline colonoscopies on occurrence of colorectal neoplasms. Clin Gastroenterol Hepatol.

[20] Morita T (2005). The metabolic syndrome is associated with increased risk of colorectal adenoma development: the Self-Defense Forces health study. Asian Pac J Cancer Prev.

[21] Sha N (2016). The evaluation of the association between the metabolic syndrome and tumor grade and stage of bladder cancer in a Chinese population. Onco Targets Ther.

[22] Ni J (2015). Metabolic syndrome is an independent prognostic factor for endometrial adenocarcinoma. Clin Transl Oncol.

[23] Rosato V (2011). Metabolic syndrome and endometrial cancer risk. Ann Oncol.

[24] Furberg AS, Thune I (2003). Metabolic abnormalities (hypertension, hyperglycemia and overweight), lifestyle (high energy intake and physical inactivity) and endometrial cancer risk in a Norwegian cohort. Int J Cancer.

[25] Bjorge T (2011). Metabolic risk factors and ovarian cancer in the metabolic syndrome and cancer project. Int J Epidemiol.

[26] El Hayek S (2016). Poly cystic ovarian syndrome: an updated overview. Front Physiol.

[27] Kubota T (2013). Update in polycystic ovary syndrome: new criteria of diagnosis and treatment in Japan. Reprod Med Biol.

[28] Hou X (2008). Risk factors for overweight and obesity, and changes in body mass index of Chinese adults in Shanghai. BMC Public Health.

[29] Organization, W.H. Weight Control and Physical Activity. Weight Control & Physical Activity. 2002: p. 1–315.

[30] Calle EE (2003). Overweight, obesity, and mortality from cancer in a prospectively studied cohort of U.S. adults. N Engl J Med.

[31] Pothiwala P, Jain SK, Yaturu S (2009). Metabolic syndrome and cancer. Metab Syndr Relat Disord.

[32] Reeves GK (2007). Cancer incidence and mortality in relation to body mass index in the Million Women Study: cohort study. BMJ.

[33] Barrett SV (2008). Does body mass index affect progression-free or overall survival in patients with ovarian cancer? Results from SCOTROC I trial. Ann Oncol.

[34] Fader AN (2009). Endometrial cancer and obesity: epidemiology, biomarkers, prevention and survivorship. Gynecol Oncol.

[35] Pavelka JC (2006). Effect of obesity on survival in epithelial ovarian cancer. Cancer.

[36] Modesitt SC, van Nagell JR (2005). The impact of obesity on the incidence and treatment of gynecologic cancers: a review. Obstet Gynecol Surv.

[37] Zhang M (2005). Body mass index in relation to ovarian cancer survival. Cancer Epidemiol Biomarkers Prev.

[38] Bonuccelli G (2010). Ketones and lactate “fuel” tumor growth and metastasis: evidence that epithelial cancer cells use oxidative mitochondrial metabolism. Cell Cycle.

[39] Bianchini F, Kaaks R, Vainio H (2002). Overweight, obesity, and cancer risk. Lancet Oncol.

[40] Kluth LA (2013). Obesity is associated with worse outcomes in patients with T1 high grade urothelial carcinoma of the bladder. J Urol.

[41] Oshakbayev KP (2014). Weight change therapy as a potential treatment for end-stage ovarian carcinoma. Am J Case Rep.

[42] Craig ER (2016). Metabolic risk factors and mechanisms of disease in epithelial ovarian cancer: a review. Gynecol Oncol.

[43] Bakhru A, Buckanovich RJ, Griggs JJ (2011). The impact of diabetes on survival in women with ovarian cancer. Gynecol Oncol.

[44] Park J (2014). Obesity and cancer--mechanisms underlying tumour progression and recurrence. Nat Rev Endocrinol.

[45] Hursting SD (2003). Calorie restriction, aging, and cancer prevention: mechanisms of action and applicability to humans. Annu Rev Med.

[46] Voutsadakis I.A.. Obesity and diabetes as prognostic factors in patients with colorectal cancer. Diabetes Metab Syndr 2016. 10.1016/j.dsx.2016.12.018 27989518

